# Transketolase-Like 1 Expression Is Modulated during Colorectal Cancer Progression and Metastasis Formation

**DOI:** 10.1371/journal.pone.0025323

**Published:** 2011-09-27

**Authors:** Santiago Diaz-Moralli, Miriam Tarrado-Castellarnau, Cristina Alenda, Antoni Castells, Marta Cascante

**Affiliations:** 1 Departament de Bioquimica i Biologia Molecular, Facultat de Biologia, Institut de Biomedicina at Universitat de Barcelona IBUB and IDIBAPS-Hospital Clinic, University of Barcelona, Barcelona, Spain; 2 Pathology Department, Hospital General Universitario de Alicante, Alicante, Spain; 3 Gastroenterology Department, Hospital Clínic, IDIBAPS, CIBEREHD, University of Barcelona, Barcelona, Spain; Thomas Jefferson University, United States of America

## Abstract

**Background:**

Transketolase-like 1 (TKTL1) induces glucose degradation through anaerobic pathways, even in presence of oxygen, favoring the malignant aerobic glycolytic phenotype characteristic of tumor cells. As TKTL1 appears to be a valid biomarker for cancer prognosis, the aim of the current study was to correlate its expression with tumor stage, probability of tumor recurrence and survival, in a series of colorectal cancer patients.

**Methodolody/Principal Findings:**

Tumor tissues from 63 patients diagnosed with colorectal cancer at different stages of progression were analyzed for TKTL1 by immunohistochemistry. Staining was quantified by computational image analysis, and correlations between enzyme expression, local growth, lymph-node involvement and metastasis were assessed. The highest values for TKTL1 expression were detected in the group of stage III tumors, which showed significant differences from the other groups (Kruskal-Wallis test, *P* = 0.000008). Deeper analyses of T, N and M classifications revealed a weak correlation between local tumor growth and enzyme expression (Mann-Whitney test, *P* = 0.029), a significant association of the enzyme expression with lymph-node involvement (Mann-Whitney test, *P* = 0.0014) and a significant decrease in TKTL1 expression associated with metastasis (Mann-Whitney test, *P* = 0.0004).

**Conclusions/Significance:**

To our knowledge, few studies have explored the association between variations in TKTL1 expression in the primary tumor and metastasis formation. Here we report downregulation of enzyme expression when metastasis appears, and a correlation between enzyme expression and regional lymph-node involvement in colon cancer. This finding may improve our understanding of metastasis and lead to new and more efficient therapies against cancer.

## Introduction

Tumor development is caused by a sequential accumulation of genetic and epigenetic changes that lead cells through a multistep process that renders healthy cells malignant [1]–[4]. Throughout this process, alterations that favor the formation of primary tumors usually differ from the adaptations required for advanced carcinogenesis [5]–[7]. Carcinogenesis onset is due to deregulation of cell-cell and cell-matrix growth inhibitory interactions and the accumulation of further genetic alterations that leads to activation of proto-oncogenes and inhibition of tumor-suppressor genes [7], [8]. After these early events, malignant transformation progresses, governed by a process of Darwinian selection during which phenotypes, rather than genotypes, are selected. This gives tumor cells an advantage over non-transformed cells. This selection of phenotypes, independent of the associated genotypes, is the basis for the high genetic heterogeneity of cancer cells, since different (epi)genetic mechanisms can converge in similar phenotypes [7], [9]. One of the main phenotypic characteristics of cancer cells is the aerobic glycolysis associated with elevated, but inefficient, ATP production, as well as high production of NADPH and acids (H^+^) such as lactate [10]. This phenomenon, known as the “Warburg effect”, was described by Otto Warburg in 1924 [11]. It forms the basis for positron emission tomography (PET), a technique that is widely used to detect alterations in glucose consumption in cancer patients. The high glycolytic rate is not justified by energetic requirements, since more than 80% of ATP synthesis in tumor cells occurs through oxidative phosphorylation, and only about 17% occurs through the Embden-Meyerhof pathway (EMP). These proportions are similar to those found in non-tumor cells [12]. On the other hand, NADPH formation is important for tumor-cell metabolism, since it protects them against oxidative stress and provides fuel for the increased rate of fatty acid synthesis characteristic of cancer cells. Finally, lactate production and microenvironment acidification give tumor cells an advantage over healthy cells, by enhancing their invasiveness and metastasis [7], [13]–[16]. Surprisingly, acidification of the environment is independent of EMP, since in glycolysis-impaired cells this phenomenon is still observable [17], [18].

In addition to aerobic glycolysis, the pentose phosphate pathway (PPP) is important for tumor metabolism, since more than 85% of the ribose necessary for nucleic acid synthesis in tumor cells is generated, directly or indirectly, from the nonoxidative branch of the PPP [19]. Therefore, the PPP lies at the basis of tumor metabolism regulation. G6PDH and TKT, which control the pathway, are enough to explain the basic metabolic features acquired by tumor cells during their transformation. Through the PPP, cells not only synthesize the nucleic acid precursors necessary to maintain the accelerated proliferation rate characteristic in cancer. G6PDH regulates the flux of the oxidative branch through which NADPH is synthesized and CO_2_ is released, leading to matrix acidification by carbonic anhydrase. The thiamine-dependent enzyme TKT shows the highest control coefficient over the non-oxidative branch of the pathway, revealing its key role in the regulation of the PPP [20], [21]. TKT has been postulated to link the PPP and the EMP in cancer cells, allowing oxygen-independent glucose degradation [22]. On the other hand, the role of TKT in tumor-cell metabolism has been underlined by reports of a significant decrease in tumor-cell proliferation following treatment with specific TKT inhibitors, both *in vitro* and *in vivo* [21]–[30]. Moreover, when TKT is activated by addition of its cofactor, thiamine, tumor growth is stimulated [31].

One TKT and two transketolase-like genes (TKTL1 and TKTL2) have been described in the human genome [32]. This finding led to examine the expression of all three enzymes in cancer cells and conclude that TKTL1 was the only one specifically upregulated in tumors [33]. Subsequent studies revealed that TKTL1 is responsible for around 60% or 70% of transketolase activity in human hepatoma and colon-cancer cells [33]–[35] showing the important role played by this isoenzyme in these tumors. It has been hypothesized that TKTL1 could generate acetylCoA for fatty acid synthesis, which would link anaerobic glucose degradation and lipogenesis [32]. Moreover, overexpression of the enzyme has been reported in several tumor cells and tissues, e.g. in colon and urothelial cancer [33], [36], gastric cancer [37], various gynecological cancers (ovarian, granulose cell of the ovary and uterine cervix) [38]–[40], breast cancer [41], [42], papillary thyroid carcinomas [43] and non-small cell lung cancer [44]. It has also been demonstrated that the specific inhibition of TKTL1 expression by shRNA triggers apoptosis and suppresses tumor growth [35]. This correlation between the overexpression of TKTL1 and tumor growth, poor survival and tumor recurrence, in addition with the transcriptional upregulation of the enzyme expression caused by promoter demethylation [45] led Smith and coworkers to suggest the enzyme as a potential proto-oncogene [46]. Furthermore, it has also been proposed that TKTL1 induces the malignant aerobic glycolytic phenotype by enhancing fructose-6-phosphate and glyceraldehyde-3-phosphate production, resulting in elevated fluxes to pyruvate and lactate [16], [46]. Nevertheless, there is a need for more detailed study of the correlation of TKTL1 with tumor progression in colorectal cancer, to elucidate its role in lymph-node affection and metastasis. Moreover, to date image quantification of enzyme expression in tissues has been performed by semiquantitative methods based on expert observation and subjective evaluation [38], [47] proving the need of an improvement in the evaluation system that allow obtaining objective results.

Here we present a new image analysis quantification method for the evaluation of immunostains that allow us to examine how TKTL1 expression varies with the progression stage in a series of colorectal carcinomas. Samples are classified in four groups or stages of progression (I to IV) according to the American Joint Committee on Cancer (AJCC) staging manual (sixth edition), which takes into account transmural extension, lymph node involvement and presence of distant metastases. Possible correlations between TKTL1 expression and tumor progression are explored, to determine its role during tumor development.

## Results

### Patients

Expression of TKTL1 was analyzed by immunohistochemistry in 63 patients with primary colorectal cancer (CRC). Demographic, clinical and tumor-related characteristics are listed in **Table S1** and **Table S2**. After a median follow-up of 49 months, 26 patients had died.

### Immunohistochemical detection of TKTL1 expression

**Figure 1:**
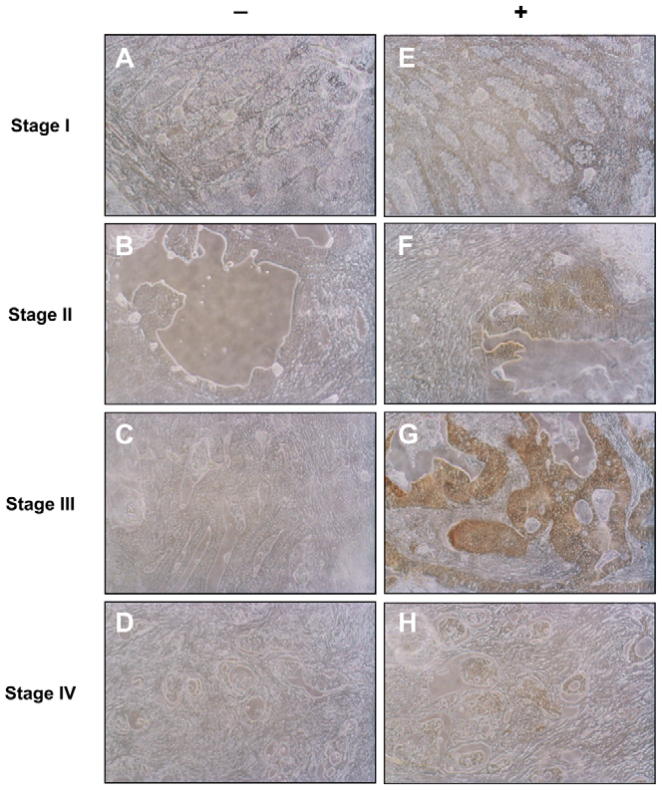
Expression of TKTL1 in colorectal tumors in different stage of progression. These color photomicrographs reveal cytoplasmatic immunohistochemical staining (brown deposits) for transketolase-like 1 (TKTL1) in colorectal tumors (original magnification ×200). In the right column (E–H) there are stained tissues at different stages of progression and in the left column (A–D) homologous areas in negative controls. Staining for TKTL1 is observable in all positive samples, indicating its expression in tumor tissues; the highest intensity is detected in stage III samples.

**Figure 2:**
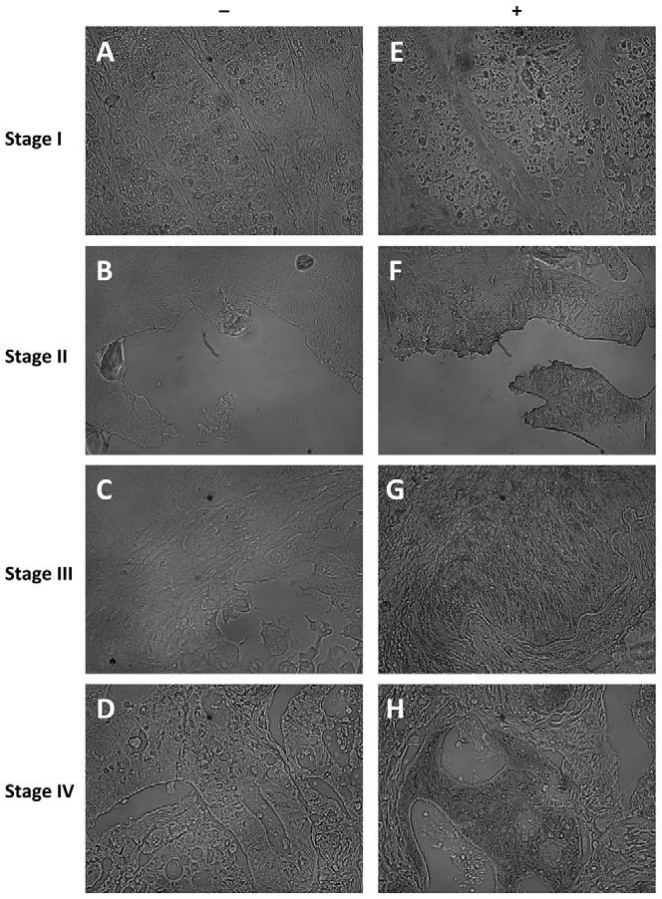
Images used for TKTL1 expression quantification. These monochromatic photomicrographs were analyzed with ImageJ software to quantify immunostaining and evaluate TKTL1 expression (original magnification ×200). In the right column (E–H) there are stained tissues at different stages of progression and in the left column (A–D) homologous areas in negative controls.

Primary tumors incubated with anti-TKTL1 antibody showed significant labeling, whereas control samples did not show any unspecific labeling (**Figure 1** and **Figure 2**). Levene and Cochrand tests did not establish that our data were normally distributed (results not shown) so we performed nonparametric tests to assess the significance of the differences. By applying Mann-Whitney U or Kruskal-Wallis tests, we confirmed that TKTL1 expression was not gender- or age-dependent (results not shown). Clinico-pathological and immunohistochemical data are summarized in **Table S1**.

### TKTL1 variations according to tumor stage

**Figure 3:**
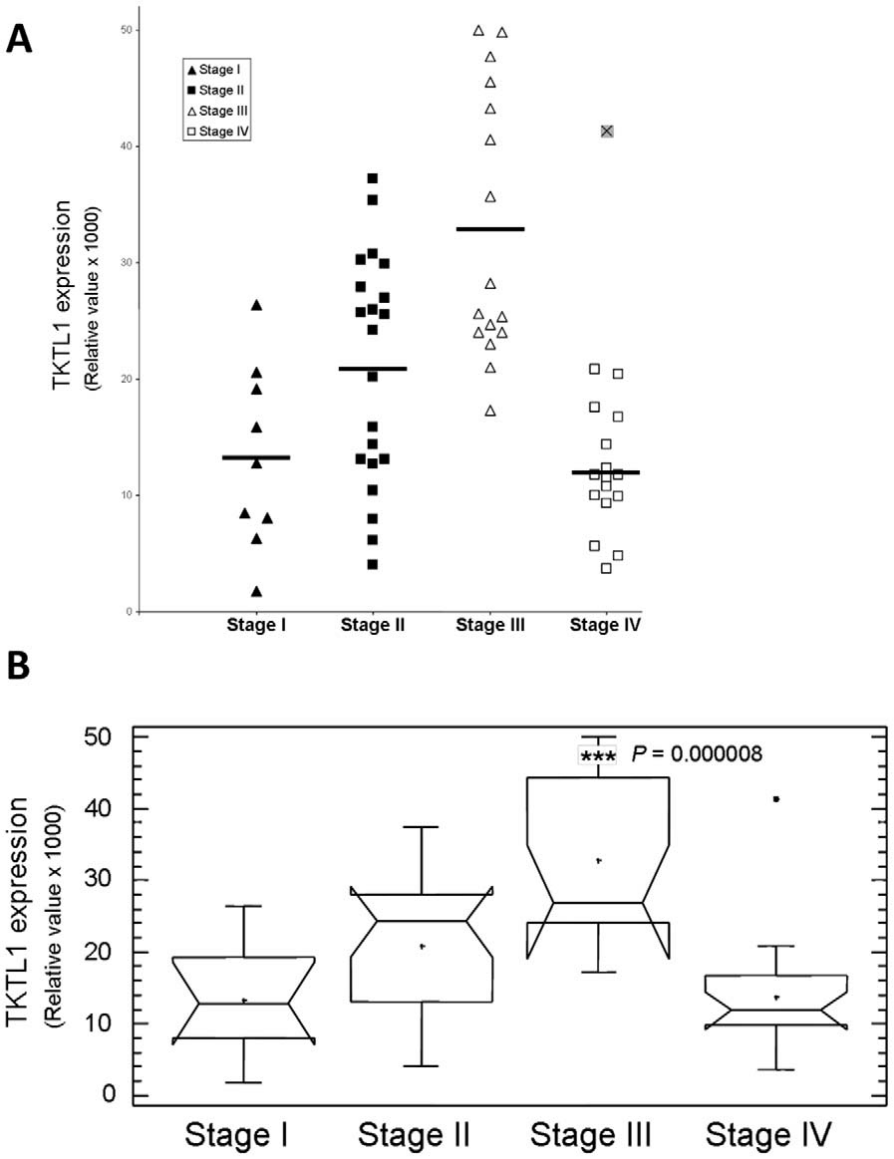
TKTL1 expression varies depending on the stage of progression of the CRC. These graphs show the expression of transketolase-like 1 (TKTL1) in the CRC tissues analyzed, divided by groups according to their stage of progression. (A) Each point corresponds to one sample. In the stage IV group the cross represents an outlier. Averages are marked by a horizontal black line in each group. (B) This box-and-whisker plot shows the differences between groups. The outlier of stage IV group is represented by a black square. Stage III group is significantly different from the rest of groups (Kruskall-Wallis test, *P* = 0.000008).

The expression of TKTL1 ranged: from 1.8 to 26.4 a.u. (mean, 13.3±7.9) in stage I tumors, from 4.0 to 37.3 a.u. (mean, 20.8±9.9) in stage II, from 17.3 to 50.0 a.u. (mean, 32.9±11.5) in stage III and from 3.7 to 41.3 a.u. (mean, 13.7±8.7) in stage IV (**Figure 3A**). These data demonstrate that expression of TKTL1 in stage III tumors was higher than at any other stage (Kruskal-Wallis test, *P* = 0.00003, **Figure 3B**). However, one sample (id. #7118) showed an abnormally highly value (41.3) in comparison with the rest of the samples of this group. The Dixon's Q-test identified this value as an outlier with a confidence level of 99.9% and, accordingly, it was excluded from the analysis. After exclusion of this sample, TKTL1 expression in stage IV tumors ranges from 3.7 to 20.8 a.u. (mean, 12.0±5.1) and expression of TKTL1 in stage III tumors become even more different (Kruskal-Wallis test, *P* = 0.000008).

TKTL1 expression in stage III tumors was significantly higher than at any other progression stage. Moreover, when considered separately, almost every individual stage showed significantly different values for TKTL1 expression from both the previous and the subsequent stage: stage I showed a tendency to be lower than stage II (Mann-Whitney U test, *P* = 0.06). Enzyme expression in stage II tumors was significantly lower than in stage III tumors (Mann-Whitney test, *P* = 0.02) and stage III values were significantly higher than those of stage IV (Mann-Whitney U test, *P* = 0.000003).

### Correlation between presence of distant metastasis and tumor TKTL1 expression

**Figure 4:**
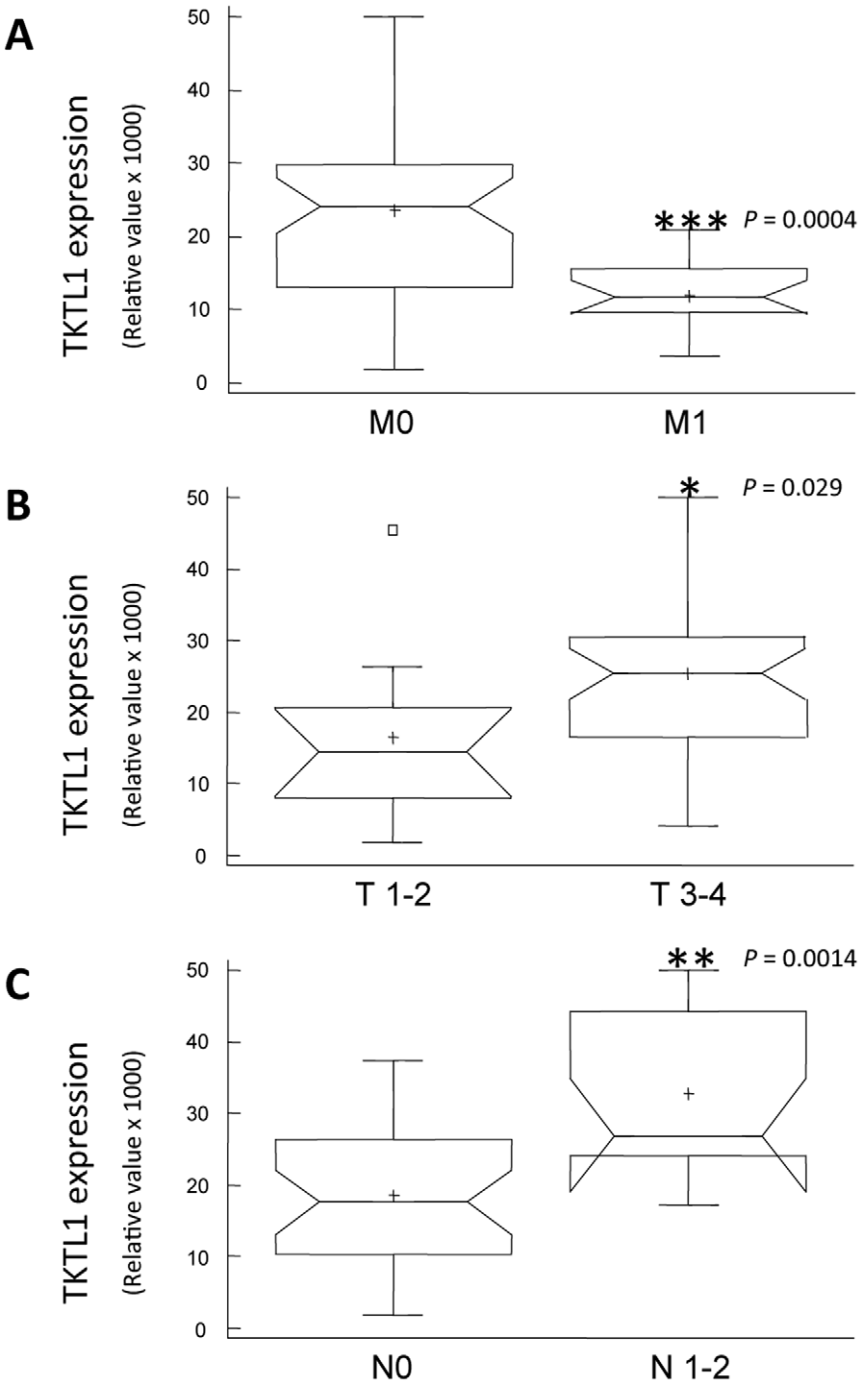
Correlation between TKTL1 expression and TNM classifications. These bar charts illustrate the correlation between transketolase-like 1 (TKTL1) expression and tumor classification values. (A) Tumor TKTL1 expression in patients who did not present with distant metastasis (M0) is significantly higher than the expression in patients who had developed metastasis (M1) (Mann-Whitney U test, *P* = 0.0004). (B) In patients who had not developed metastasis, expression of TKTL1 tends to be lower in early stage tumors with respect to transmural invasion (T1–2) than in more advanced tumors (T3–4) (*P* = 0.029, Mann-Whitney U test). (C) Lymph node invasion correlates with an increase in TKTL1 expression in the primary tumor (Mann-Whitney U test, *P* = 0.0014).

A strong decrease in primary tumor TKTL1 expression was observed in patients who presented with distant metastasis. Indeed, samples from patients without metastases (M0) exhibited expression values between 1.8 and 50.0 a.u. (mean 23.5±12.4) while values for samples from patients who developed distant metastasis (M1) ranged from 3.7 to 20.8 a.u. (mean 12.0±5.1) (Mann-Whitney U test, *P*-value = 0.0004) (**Figure 4A**). Taking into account the differences between M0 (non-metastatic) and M1 (metastatic) tumors, we performed the following comparisons only for samples from the M0 group.

### TKTL1 involvement in tumor progression

With respect to transmural progression, early stage tumors (T1 and T2) presented TKTL1 expression values between 1.8 and 45.5 a.u. (mean 16.5±12.6), while expression of the enzyme in more advanced primary tumor (T3 and T4) ranged from 4.0 to 50.0 a.u. (mean 25.5±11.8). These data indicate a slight increase in TKTL1 expression when local development of tumor increases (Mann-Whitney U test, *P* = 0.029, **Figure 4B**).

TKTL1 expression in samples without regional lymph-node involvement (N0) ranged from 1.8 to 37.3 a.u. (mean 18.6±9.8), whereas the corresponding values for those with regional lymph-node involvement (N1 and N2) ranged from 17.3 to 50.0 a.u. (mean 32.9±11.5) (Mann-Whitney U test, *P* = 0.0014) (**Figure 4C**).

### TKTL1 expression and survival

**Figure 5:**
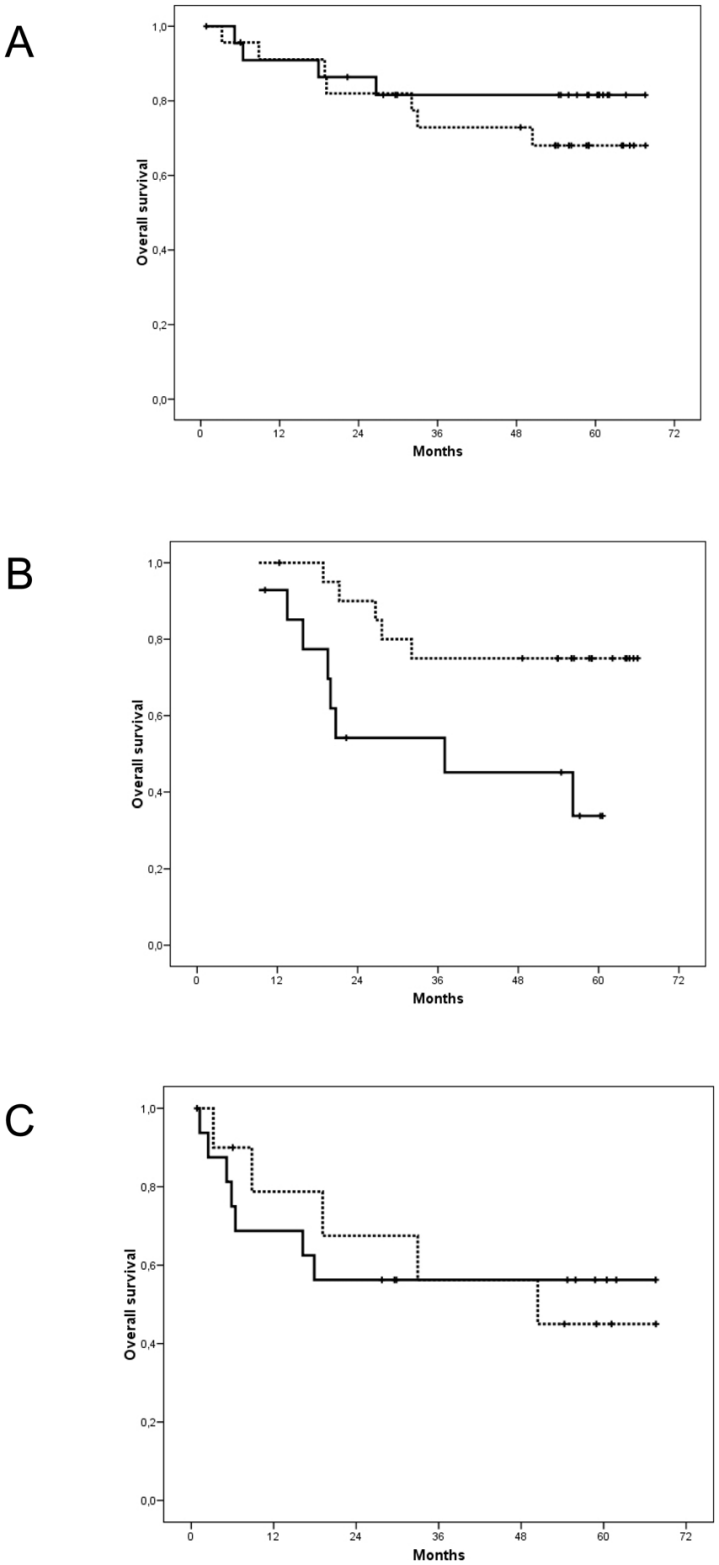
Correlation between TKTL1 expression and patient survival. (A) Kaplan-Meier plot correlating TKTL1 expression and survival in patients with non-metastatic CRC. (B) Kaplan-Meier plot correlating TKTL1 expression and survival in untreated patients. (C) Kaplan-Meier plot correlating TKTL1 expression and survival in patients with CRC treated with 5-fluoroacyl. Solid line represents those with TKTL1 expression below the mean value, and dotted line represents those with TKTL1 expression above the mean value for each group.

Univariate Kaplan-Meier analysis did not reveal significant association between TKTL1 staining and survival in CRC tissue samples (Mantel-Cox log-rank test, *P* = 0.37) (**Figure 5A**). On the other hand, when treatment is considered differences arise. Our data reveal a significant association between TKTL1 expression and survival in patients treated with 5-fluoroacyl (Mantel-Cox log-rank test, *P* = 0.017) (**Figure 5B**), this correlation is not observed in untreated patients (Mantel-Cox log-rank test, *P* = 0.993) (**Figure 5C**).

### Validation of the image analysis based immunostaining quantification method

**Figure 6:**
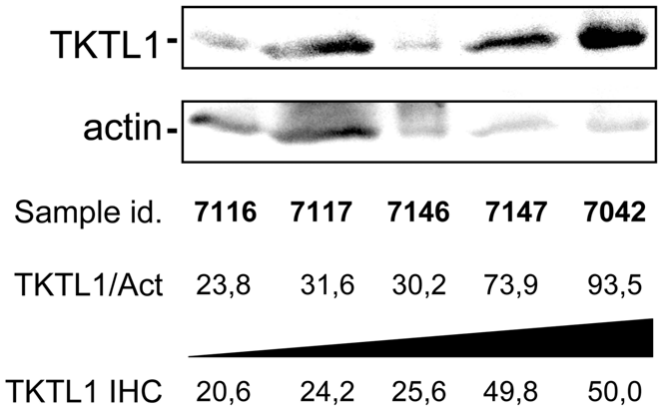
Validation of immunostain quantification through western blot analysis. TKTL1 protein and actin were analyzed by western blot using specific monoclonal antibodies. TKTL1/Act represents the quantitative analysis of TKTL1 corrected by actin. TKTL1 IHC are the expression values of TKTL1 determined by immunohystochemical analysis. All the 5 colon cancer sample homogenates analyzed show similar expression values for TKTL1 in both analyses.

For the validation of the image analysis quantification method, 5 samples belonging to stage I, II and III of progression, and with different TKTL1 expression level were analyzed by western blot. Immunodetection of TKTL1 and actin as loading control showed a clear correlation between TKTL1 expression determined by western blot and by immunohystochemical analysis (**Figure 6**).

## Discussion

The expression of TKTL1 has been detected and correlated with several kinds of cancer so far. Most of the authors reporting these correlations follow a protocol analogous to that described in the current paper, using the same antibody and kit for the immunological staining. However, to date the evaluation of immunostaining was inaccurate, since it has been performed applying a semiquantitative system based on the assignment of scores by observers. The current manuscript describes a new and more objective system to quantify the staining through computational image analysis. This technique allows obtaining quantitative data from immunohystochemical staining leading to a more accurate evaluation of enzyme expression. Therefore the reported method is revealed as a really powerful tool for immunohystochemical quantification of protein expression that offers objective numerical data instead of arbitrary scores.

Our results provide evidence that TKTL1 expression in primary colorectal cancer is strongly correlated with tumor progression. The capacity to degrade glucose under anaerobic conditions is critical to tumor growth, especially as the primary tumor expands and the internal cells are carried away from the basement membrane and their oxygen supply. In this situation, TKTL1 overexpression confers an advantage on malignant cells and allows them to grow faster. Our data show that TKTL1 expression correlates strongly with local progression (T) and regional lymph node affection (N). This is the first time that the correlation between TKTL1 expression and N classification has been reported in CRC. Moreover, we show that the correlation is stronger than that between TKTL1 expression and T classification (**Figure 4B and C**), which indicates that the effect of the enzyme increases as the tumor progresses. This behavior is consistent with the finding that overexpression of TKTL1 favors the anaerobic metabolization of glucose, increasing lactate production and inducing the environment acidification that enhances local invasiveness. Although our data do not reveal any significant association between tumor TKTL1 staining and patient survival, the univariate Kaplan-Meier analysis plotted in **Figure 5A** would tend to suggest that a larger sample might reveal a significant correlation. Furthermore, although the number of samples is small, correlation between TKTL1 expression and survival in patients undergoing chemotherapy seems clear. The observed correlation suggest that 5-fluoroacyl may be efficient in treating tumors with high levels of TKTL1, those with a high rate of local progression (**Figure 5B**). However, chemotherapy does not show any effect on survival of patients with tumors containing low levels of the enzyme, the most metastatic ones (**Figure 5C**). These data suggest that TKTL1 levels could be a potential biomarker for predicting tumor response to chemotherapy.

The current study also shows another interesting finding, the highly significant decrease in TKTL1 expression observed when comparing non-metastatic patients (M0) with metastatic ones (M1). This downregulation of the enzyme when metastasis occurs, like the correlation with the regional lymph-node involvement, has not been reported before. This is the first time that the expression of TKTL1 has been correlated with tumor staging and metastasis formation in CRC. Interestingly, similar behavior has been described in the incidence of mutated *ras* in CRC: the incidence of mutation increases from ∼7% in early adenoma to ∼55% in intermediate, and to ∼60% in late carcinomas, but decreases to ∼45% in invasive carcinomas, indicating an implication of this mutation in cell aggressiveness but not in metastasized cells [7], [48].

The regulation of TKTL1 expression in tumor cells is largely unexplored, but it has been proposed that it could be upregulated in response to hypomethylation [46]. The mechanism responsible for global demethylation in cancer cells remains unknown, although Ras family members have been implicated in the regulation of methylation [49]–[52]. In most cases, Ras-signaling has been related to hypermethylation and inhibition of tumor suppressor genes. However, it has also been related to demethylation in some studies [53]–[56]. Here we propose a new role for Ras-signaling in tumor transformation that consists of the activation by hypomethylation of different oncogenes, including TKTL1 in CRC.

Notably, Ras superfamily members have been implicated in the regulation of aerobic glycolysis by increasing glucose uptake and 6-phosphofructo-2-kinase/fructose-2,6-bisphosphates 3 (PFKFB3) expression [57]–[59]. PFKFB3 activity causes the accumulation of fructose-2,6-bisphosfate (F26bP) and the subsequent upregulation of 6-phosphofructo-1-kinase (PFK-1) activity, which finally leads to elevated lactate production. The metabolic substrate for PFKFB3 and PFK-1 reactions, which are essential for lactate production, is fructose-6-phosphate (F6P), one of the products of TKTL1. Furthermore, TKTL1 overexpression favors normoxic stabilization of the malignancy-promoting transcription factor hypoxia-inducible factor-1α (HIF-1α) [16] and the upregulation of downstream glycolytic enzymes such as glucose transporter 1 (GLUT1) and PFKFB3 [16], [60]. As we have seen, TKTL1 plays an important role in pyruvate and lactate production via its final products, fructose-6-phosphate and glyceraldehyde-3-phosphate. Therefore, since pyruvate and lactate regulate hypoxia-inducible gene expression and inactivate HIF-1α decay [61], [62], TKTL1 overexpression may stimulate the accumulation of HIF-1α in a hypoxic-independent manner and promote the expression of HIF-1-regulated genes involved in glycolytic metabolism, angiogenesis and cell survival.

In short, during carcinogenesis onset, mutations in Ras members could accumulate and lead to TKTL1 promoter demethylation and activation of its expression. This phenomenon would be selected because TKTL1 activity would permit transformed cells to consume glucose in the absence of oxygen, produce lactate and acidify their microenvironment, thus strengthening its invasiveness. TKTL1 may also lead to hypoxic-independent HIF-1α stabilization and the subsequent overexpression of several glycolytic and angiogenic genes, providing a suitable environment for tumor progression. Regarding metastasis formation two possible mechanisms could explain observed data: i) TKTL1 overexpression not only lead to tumor progression, but also could enable metastasis formation, and when metastasis is established, TKTL1 is no longer necessary, and like the incidence of mutated *ras*, it decreases. ii) TKTL1 is necessary for tumor progression in situ and local invasiveness, but tumors that do not overexpress the enzyme are incapable of growing in its local environment and induce the metastatic behavior as a tumor survival strategy through a Darwinian selection process.

This report sheds light on the role of TKTL1 in tumor progression, and aims to be the first step towards a new approach to the study of metastasis and cancer therapy.

## Materials and Methods

### Ethics Statement

Ethics approval for the study was obtained from the ethics committee at the Hospital Clínic of Barcelona, written informed consent was obtained from all patients and all clinical investigation have been conducted according to the principles expressed in the Declaration of Helsinki.

### Patients

In the current study, 46 men and 17 women (70±11 years old) with colorectal carcinoma (CRC) that underwent surgery between November 2000 and October 2001 were included. According to TNM classification of colon and rectal cancer of American Joint Committee on Cancer (AJCC), 9 patients presented with stage I tumors, 21 with stage II, 16 with stage III and 17 with stage IV.

All patients were recruited at the Gastroenterology Department of the Hospital Clínic of Barcelona, and were part of the EPICOLON project, a prospective, multicenter, nation-wide, population-based study was aimed at establishing the incidence and characteristics of inherited and familial colorectal cancer forms in Spain [63]. In this project, all newly diagnosed CRC patients in any participating center during one-year period were included in the study.

After surgical resection, patients underwent standard therapeutic and follow-up measures according to recommended guidelines. Indeed, postoperative adjuvant treatment with 5-fluorouracil and leucovorin was routinely given to patients with stage II and III tumours, and radiation therapy was indicated in patients with rectal cancer. Postoperative surveillance consisted of medical history, physical examination, and laboratory studies including serum carcinoembryonic antigen (CEA) levels every three months, abdominal ultrasonography or computed tomography every six months, and chest radiograph and total colonoscopy once a year. Furthermore, all tumor recurrences detected during the follow-up were histologically confirmed.

### Immunohistochemical staining

Immediately after surgical resection, tumors were snap-frozen and kept in liquid nitrogen. To perform the staining, CRC sections were obtained using vibrotom cuts, were desiccated to prevent degradation and kept at ambient laboratory conditions until use.

Colorectal cancer specimens were cut in sections of 2 to 5 µm, placed on slides and fixed with paraformaldehyde. The slides were hydrated by rinsing them in decreasing concentrations of ethanol. For antigen unmasking samples were heated to 65°C aprox. in 10 mM sodium citrate buffer (pH 6.0) for 5 min. After rinsing in destilled H_2_O, inhibition of endogenous peroxidase was performed with a 10 min incubation with 3% H_2_O_2_. Slides were washed with PBS and incubated with 3% BSA in PBS for 15 min to block unspecific staining. Afterward, sections were incubated with a mouse monoclonal anti-TKTL1 antibody (clone JFC12T10) described previously by Coy [32] at a concentration of 4 µg mL^−1^ for 60 min in a humidified chamber at room temperature. Subsequently, slides were washed with PBS, incubated with biotinylated anti-mouse immunoglobulins (Biotinylated Link, LSAB+-kit, DakoCytomation, Hamburg, Germany) for 25 min and, after a new wash with PBS, treated with streptavidin-peroxidase (Streptavidin-HRP, LSAB+-kit, DakoCytomation, Hamburg, Germany) for 25 min more. Finally samples were incubated with 3-3′-diaminobenzidine (DAB+Chromogen, DakoCytomation, Hamburg, Germany) for 20 min at room temperature to obtain the staining.

TKTL1 expression was evaluated with image analysis using a LEICA DM 4000 B microscope (Leica Microsystems, Germany), a monochromatic IEEE-1394 CFW-1312M camera (Scion Corporation, Frederik, MD, USA) and the public domain NIH Imaging software program ImageJ, available via internet from URL: http://rsbweb.nih.gov/ij/. The intensity was measured as “relative intensity/area” and quantified by interpolation into a calibration curve plotted using a grey scale. For each sample 3 or 4 pictures from different areas were captured and the staining of a homolog area of a negative control, incubated with 3% BSA in PBS without anti-TKTL1 antibody, was subtracted. Values are presented as “Relative value×1000” arbitrary units (a.u.). This kind of informatics quantification of the staining requires 8-bit monochromatic images (**Figure 2**) and quantifies grey intensity within a calibration scale [64], [65] giving an evaluation of the TKTL1 expression that allows much more objective comparisons between samples than classifications by arbitrary scores.

### Protein Extraction

Total protein was purified from 6 to 19 mg of frozen surgical specimens. Samples were sonicated at 4°C with a titanium probe in lysis buffer containing 20 mM HEPES pH 7.5, 10% (v/v) glycerol, 0.4 M NaCl, 0.4% (v/v) Triton X-100, 10 mM EGTA, 5 mM EDTA, 25 mM NaF, 25 mM Na β-glycerophosphate, 1 mM DTT, 1× Protease Inhibitors, 0.4 mM Pefabloc SC and 20 µg/ml Pepstatin. After sonication samples were centrifuged at 4°C and 16,000 rcf for 20 minutes. Protein concentration in the supernatants was measured using the bicinchoninic acid (BCA) assay kit (Pierce, Rockford, IL, USA) according to manufacturer instructions.

### Western Blotting

Western blotting was performed by loading 30 µg of protein on a 10% SDS-polyacrylamide gel and transferred to a polyvinyl nitrocellulose transfer membrane (Bio-Rad Laboratories, Richmond). The membrane was blocked by incubation in TBS buffer (20 mM Tris, pH 7.5 and 132 mM NaCl) containing 0.1% of Tween and 5% of dry milk, for 1 h, at room temperature and washed three times with TBS-0.1% Tween. Then, the membrane was blotted with 0.2 µg/mL mouse monoclonal anti-TKTL1 antibody (clone JFC12T10) overnight at 4°C. The blot was washed three times with TBS-0.1% Tween and incubated with HRP-conjugated rabbit anti-mouse secondary antibody for 1 h at room temperature. After secondary antibody incubation, the membrane was washed again three times with TBS-0.1% Tween. The blot was developed by enhanced chemiluminiscence using a chemiluminiscence ECL Western Blotting Detection Kit Reagent and detected using LAS-3000 imaging system (Fuji Photo Film (Europe) TK Tilburg, The Netherlands). To verify similar protein loading, after TKTL1 detection the membrane was washed three times with TBS-0.1% Tween and incubated with mouse monoclonal anti-Actin antibody (MP Biomedicals, Eschwege, Germany) according to manufacturer instructions. Secondary antibody incubation and blot development were performed as described for TKTL1.

### Statistical Analyses

All results are expressed as means ± SD. Statistical analysis was performed using Statgraphics software (Statgraphics.net, Madrid, Spain). Outliers were identified using Dixon's Q-test, and differences between groups were analyzed by the Mann-Whitney *U*-test when two populations were compared and by the Kruskal-Wallis test when more than two populations were compared. Statistical significance was set at *P*<0.05, and statistical trend was defined as *P*<0.1. Probability of patient survival was studied by the univariate product-limit method of Kaplan-Meier.

## Supporting Information

Table S1 **Clinico-pathological and immunohistochemical data.** Id, arbitrary number used to preserve the privacy of patients; a.u., arbitrary units; M, male; F, female.
(XLS)

Table S2 **Demographic, clinical and tumor-related characteristics of patients included in the study (n = 63).** ^1^Expressed as mean ± standard deviation; ^2^All four cases correspond to loss of MLH1 protein expression; ^3^All chemotherapeutic regimens included 5-fluoruracil, 5 of them in combination with oxaliplatin and 2 in combination with irinotecan; ^4^Expressed as median (range).
(DOC)
