# Supplementary material for: Transketolase-Like 1 Expression Is Modulated during Colorectal Cancer Progression and Metastasis Formation
Source: PLoS One. 2011 Sep 27;6(9):e25323. doi: 10.1371/journal.pone.0025323 (PMC3181277; doi:10.1371/journal.pone.0025323)
Supplement: Table S2 — Demographic, clinical and tumor-related characteristics of patients included in the study (n = 63). 1Expressed as mean ± standard deviation; 2All four cases correspond to loss of MLH1 protein expression; 3All chemotherapeutic regimens included 5-fluoruracil, 5 of them in combination with oxaliplatin and 2 in combination with irinotecan; 4Expressed as median (range). (DOC) [file pone.0025323.s002.doc]

| Age (years)^1^ |  | 69.5 ± 11.4 |
| --- | --- | --- |
| Gender – no. (%) | Male | 45 (72.6) |
|  | Female | 17 (27.4) |
| Family history of colorectal cancer – no. (%) |  | 10 (16.1) |
| Fulfillment of revised Bethesda guidelines – no. (%) |  | 14 (22.6) |
| Tumor location – no. (%) | proximal to splenic flexure | 19 (30.6) |
|  | distal to splenic flexure | 43 (69.4) |
| TNM tumor stage – no. (%) | I | 9 (14.5) |
|  | II | 21 (33.9) |
|  | III | 16 (25.8) |
|  | IV | 16 (25.8) |
| Tumor size (mm)^1^ |  | 43 ± 20 |
| Differentiation degree – no. (%) | well | 6 (9.7) |
|  | moderate | 51 (82.2) |
|  | poor | 5 (8.1) |
| Mucinous carcinoma type – no. (%) |  | 10 (16.1) |
| Mismatch repair deficiency – no. (%)^2^ |  | 4 (6.5) |
| Synchronous colorectal cancer – no. (%) |  | 5 (8.1) |
| Synchronous colorectal adenoma – no. (%) |  | 23 (37.1) |
| Surgical treatment – no. (%) | right colectomy | 16 (25.8) |
|  | left colectomy | 6 (9.7) |
|  | sigmoidectomy | 22 (35.5) |
|  | anterior resection | 9 (14.5) |
|  | total colectomy | 6 (9.7) |
|  | abdominoperineal resection (Miles) | 3 (4.8) |
| Chemotherapy – no. (%)^3^ |  | 35 (56.5) |
| Length of follow-up (months)^4^ |  | 49 (40-53) |
